# International workshop report on “Animal resilience and organismal response to environmental change: insights from basal metazoans”, Tutzing (Germany), 22–25 September 2025

**DOI:** 10.1186/s12983-025-00592-0

**Published:** 2026-01-25

**Authors:** Kim L. de Luca, Yamini Ravichandran, Melanie Dörr, Christian R. Voolstra

**Affiliations:** 1 Helmholtz Institute for Functional Marine Biodiversity at the University of Oldenburg (HIFMB), Oldenburg, Germany; 2 Alfred Wegener Institute, Helmholtz Center for Polar and Marine Research (AWI), Bremerhaven, Germany; 3 Department of Biochemistry, University of Geneva, Geneva, Switzerland; 4 Department of Biology, University of Konstanz, Constance, Germany

**Keywords:** Basal metazoans, Animal resilience, Environmental change, Cnidarian biology, Regeneration and plasticity, Nervous system evolution, Host–microbe interactions

## Abstract

The 2025 Tutzing Workshop, held at the Evangelische Akademie on the shores of Lake Starnberg, continued a long tradition of highly integrative meetings focused on the biology and evolution of basal metazoans. The meeting was organized by Christian R. Voolstra (University of Konstanz, Germany) and Ulrich Technau (University of Vienna, Austria), with kind support from the German Research Foundation (DFG). Building on the successful 2023 event, this year’s symposium brought together close to 100 participants from Europe, North America, Asia, and Australia, representing newest research and scientific insight ranging from molecular evolution and functional genomics to ecology, developmental biology, and symbiosis. The central theme “Animal resilience and organismal response to environmental change: insights from basal metazoans” reflects an ongoing effort to leverage early-branching animals such as cnidarians (hydrozoans, anemones, jellyfish, corals), sponges, and ctenophores to address fundamental questions about the origins of multicellularity, the mechanisms of tissue regeneration, and the processes by which organisms adapt to environmental change. The symposium was structured around thematic sessions, poster presentations, roundtable discussions, and an invited keynote lecture. Scientific highlights included new genome assemblies, advances in single-cell transcriptomics, insights into epigenetic regulation and transposable element activity, as well as exciting discoveries about nervous system evolution, biomechanics of tissue regeneration, and immune responses in cnidarians. Beyond the empirical advances, the meeting fostered interdisciplinary discussion and outlined clear priorities for future collaborative research.

## Main text

The Tutzing Workshop pursued three primary objectives: (i) synthesis of current knowledge—to provide a state-of-the-art overview of research into the evolutionary and functional biology of basal metazoa, especially regarding resilience, regeneration, and complexity; (ii) integration of approaches—to bridge molecular and genomic perspectives with organismal and ecological scales, linking laboratory experiments to natural history observations; (iii) formation of interdisciplinary collaborations—to create an international dialogue that will facilitate comparative projects, standardized protocols, and shared infrastructures, ensuring that the basal metazoan community continues to drive fundamental discoveries with broad evolutionary significance [1, 2]. The meeting began with words of welcome from the organizers, emphasizing both the historic setting of Tutzing and the pressing need to connect evolutionary biology with modern functional and ecological questions. The meeting covered a total of 7 sessions: (1) Evolutionary and Functional Genomics, (2) Cell Biology, (3) Neural Development, (4) Cell Type Evolution, (5) Pattern Formation, (6) Stem Cell Differentiation and Regeneration, and (7) Ecology and Symbiosis.

In the session “Evolutionary and Functional Genomics”, several talks dealt with genome dynamics in *Hydra* and other cnidarians. Genomic atavisms and conserved synteny (Oleg Simakov, University of Vienna, Austria) explored why some animal lineages preserve deep syntenic blocks while others undergo large-scale rearrangements. The provocative hypothesis was that genome expansion via ancient TE families can stabilize enhancer-promoter configurations, preserving ancestral genome topologies. This framework termed “evolutionary genome topology” provides a new lens for understanding genome evolution and explains why some species expand, whereas others compact their genomes over evolutionary time [3]. A presentation by Sanjeev Galande (Shiv Nadar University, India) about dynamic RNA methylation demonstrated how epitranscriptomic regulation contributes to cell fate decisions in *Hydra* [4]. Using single-cell RNA-seq, the team showed that components of the m6A methylation machinery are enriched in differentiated cell populations. Functional assays revealed that disruption of m6A impaired *Hydra* head regeneration, suggesting that this modification system originally evolved as a pro-differentiation mechanism. This provided an important link between epigenetic regulation and regeneration. Closing the session, Robert Steele (UC Irvine, USA) reported the discovery and genomic characterization of *Hydra lirosoma,* a novel clade restricted to blackwater swamps such as the Okefenokee (~ 7,000 years old) and Great Dismal Swamp (~ 12,000 years old). Genome size analyses revealed a significantly smaller genome than other brown *Hydra* species. An open question remains as to how the species *Hydra lirosoma* evolved and where its origins lie. The restricted distribution and ecological specialization of *Hydra lirosoma* underscore the importance of cryptic diversity in *Hydra* and its conservation.

The “Cell Biology” session showcased several technological advances in basal metazoans. Noriko Funayama (Kyoto University, Japan) presented a method for exogenous gene expression in sponges through random introduction of fluorescent proteins. Tagging proteins with known (sub)cellular localizations enabled high-resolution live imaging of cell morphology and migration. This powerful gene introduction tool opens new avenues for functional research in sponges to better understand animal development and stem cell systems. Claudia Tortiglione (Institute of Applied Sciences and Intelligent Systems, Italy) bridged biology and materials science by generating “bionic” *Hydra*: animals that become conductive by taking up an organic compound [5]. Combined with a custom *Hydra*-on-chip configuration, bioelectrical activity was reliably recorded during spontaneous and stimulated behavior. This experimental setup opens new avenues for the study of neural signaling and electrophysiology. Together, the session exemplified how increasingly sophisticated molecular and engineering tools are being developed and applied to non-bilaterian model systems.

The session “Neural Development” addressed fundamental questions about the origin and diversification of nervous systems, with several talks highlighting single-cell and transgenic approaches. Abhishek Mishra (University of Vienna, Austria) described the identification of more than 20 distinct neural cell types in *Nematostella vectensis*, using single-cell RNA-seq. Transgenic reporter lines were being generated to validate these findings and map neural connectivity. This work positions *Nematostella* as a powerful system for studying neuronal complexity in early animals. Single-cell transcriptomics further showed how neurogenic programs and spatial patterning mechanisms resemble those of bilaterians. The group of Michael Layden (Lehigh University, USA) traced the temporal axis of neuronal fate specification, supporting the idea that bilaterian nervous system innovations were built on deeply ancestral cnidarian frameworks [6]. A molecular, spatial, and regulatory atlas of the *Hydra vulgaris* nervous system, presented by Celina Juliano (UC Davis, USA), is the most comprehensive characterization to date, identifying eight neuron types resolved into 15 subtypes [7]. Trajectory inference, ATAC-seq, and identification of transcription factors hereby provide a regulatory context for neuronal differentiation during development, homeostasis, and regeneration in *Hydra vulgaris*. The datasets were made publicly available via an interactive portal named “OpenHydra” (https://openhydra.org), underscoring the field’s commitment to sharing of resources. Together, these talks highlighted the emerging consensus that cnidarians harbor considerable molecular complexity, making them ideal systems for understanding the evolutionary origins of nervous systems.

The session “Cell Type Evolution” explored how conserved and novel molecular mechanisms shape the diversification of cell types. Yehu Moran (Hebrew University of Jerusalem, Israel) presented an ancestral and complex antiviral program in cnidarians, with compelling evidence from *Nematostella* that viral-mimic stimulation activates specialized immune cell populations characterized by distinct transcriptional signatures and increased phagocytosis [8]. Comparative analyses showed conservation of immune expression profiles with stony corals, suggesting that innate antiviral programs were already sophisticated in the course of evolution in early metazoans [9]. Functional assays using CRISPR/Cas9 mutants revealed homologs of vertebrate interferon pathways alongside RNAi-like components, a striking example of deep evolutionary mosaicism. The discussion highlighted how these findings blur the traditional division between “primitive” and “advanced” immune systems, underscoring the need to reassess the evolutionary origins of antiviral defense. Alexander Klimovich (CAU Kiel, Germany) presented complementary insights into the emergence of neuronal diversity in *Hydra*, focusing on the role of lineage-restricted genes (LRG). Combining transcriptomics, phylogenomics, and functional assays, they found pronounced LRG expression in the neuronal lineage, with roles in cnidarian locomotion and feeding. The study highlights how taxa-specific genes complement conserved regulation to generate behavioral complexity.

Emerging and established concepts governing body axis formation, tissue morphogenesis, and tissue patterning during development and regeneration of basal metazoans were covered in the “Pattern Formation” session. Biophysical studies on tissue morphogenesis highlighted the important role of mechanical cues and showed how basal metazoan research has attracted interdisciplinary researchers. Yamini Ravichandran (University of Geneva, Switzerland) reported that the morphogenetic role of tissue-scale actin organization and discontinuities defined as actin asters are responsible for shaping the emerging head during tissue regeneration in *Hydra* [10]*.* By changing the topology of the animal to a toroid, she demonstrated how the absence of actin asters inhibited head regeneration in the animal, establishing tissue-scale actin organization as a mechanical morphogen in *Hydra*. In *Nematostella vectensis,* Ulrich Technau (University of Vienna, Austria) reported that upon the removal of the transcription factor Twist, striking morphogenetic defects defined as a “neoplasm” were observed in adult polyps [11]. These findings imply that Twist integrates major signaling pathways to regulate tentacle patterning and maintain spatial tissue organization. Masha Broun (Tannenbaum Research Institute, Canada) explored the mechanisms driving ectopic foot induction and the broader signaling interactions that shape axial fate specification in *Hydra*. Her work identified Sox3—a cnidarian-specific member of the Sox transcription factor family, expressed in a pattern complementary to FoxA—as a key regulator that defines the FoxA/Sox3 boundary through transcriptional repression. More broadly, such mutually repressive gene pairs function as a general mechanism to sharpen and refine expression domains, a principle that also appears to govern axial patterning processes in *Nematostella*.

The session “Stem Cell Differentiation and Regeneration” explored how cnidarians rebuild complex structures through cellular reorganization and stem-cell plasticity. Yuichiro Nakajima (University of Tokyo, Japan) presented their work on the colonial emerging jellyfish model *Cladonema pacificum*, showing that injured polyps undergo striking organism-level reorganization rather than direct tissue replacement [12]. Regenerating polyps form cyst-like aggregates through stolon emergence and body reconstruction, accompanied by injury-induced expansion of stem-like cells. These findings suggest that cellular reorganization might represent a primordial mode of regeneration among basal metazoans. Building on the concepts of cellular plasticity and organismal self-organization, Uri Frank (University of Galway, Ireland) and colleagues examined whole-body regeneration from dissociated adult organism cell aggregates in *Hydractinia symbiolongicarpus* [13]. They demonstrated the regeneration of complete individuals guided by WNT and sphingosine-1-phosphate signaling, which are nearly exclusively derived from i-cell progeny rather than recycled somatic cells, as shown in other hydrozoans. Remarkably, although these i-cell progeny aggregates are generated from embryonic and not adult cells, they can directly form adult polyps, bypassing the larval planula stage.

A highlight of the “Ecology and Symbiosis” session was the demonstration that lunar cycles directly influence skeletal development in corals, presented by Isabel Martinez-Rugerio (HIFMB Oldenburg, Germany). By exposing only a single polyp within a larger colony fragment to moonlight, the study showed that growth signals propagate to adjacent polyps, resulting in coordinated formation of skeletal dissepiments across modules. The findings provide experimental evidence for inter-polyp communication as a mechanism enabling colonies to synchronize skeletal architecture with environmental cues. Addressing intervention-oriented strategies, Melanie Dörr (University of Konstanz, Germany) reported a standardized screening framework for identifying probiotic bacterial strains that enhance cnidarian thermal tolerance [14]. By integrating bacterial isolate libraries with acute heat stress assays, the team identified multiple bacterial isolates that increased host thermal tolerance. This systematic approach provides a scalable screening framework to inform active intervention and elucidate the mechanistic underpinnings of host-microbe interactions [15]. Jinru He’s presentation (CAU Kiel, Germany) emphasized the developmental consequences of altered host–microbe associations. Germ-free *Hydra* displayed marked developmental defects, including loss of budding capacity, which were restored by recolonization with native microbiota. Introduction of specific symbionts could induce tumorigenesis, indicating that microbial community composition has direct consequences for host development. Most notably, single-cell RNA-seq and ATAC-seq analyses revealed that microbial absence reshaped stem cell regulatory trajectories, providing mechanistic evidence that microbiota modulate stem cell and developmental programs [16].

An overall highlight of the workshop was the evening keynote by Cassandra Extavour (Harvard University, USA), which provided a broad evolutionary perspective on the origins of novel genes in animals [17]. Extavour presented evidence that chimeric genes can arise through the fusion of metazoan and non-metazoan gene fragments, often mediated by horizontal sequence transfer. Functional analyses across multiple species confirmed that many of these genes are expressed and may contribute to diverse physiological processes. This model of genetic novelty underscores gene fusion and transfer as underappreciated engines of evolutionary innovation in animal lineages. A similar highlight was remembering the legacy of Hans Bode, whose pioneering work on *Hydra* reaggregation and positional information first revealed the self-organizing capacity of cnidarian tissues. Beyond his scientific impact, Hans played a central role in shaping the *Hydra* community and the Tutzing meetings, fostering an open, collaborative spirit that continues to define this field. His passing earlier this year was deeply felt, and Charles David movingly reflected on Bode’s lasting influence during a tribute at this year’s meeting. The continued exploration of the principles he championed stands as a fitting testament to his enduring legacy.

Besides the presentations, over 30 posters provided a vibrant platform to discuss additional cutting-edge research. Topics included cnidarian microbiomes, comparative regenerative biology, and advances in genome annotation pipelines. For instance, Lukas Becker’s (Heinrich Heine University Düsseldorf, Germany) work on host-driven metabolic specialization of *Curvibacter*, a prominent *Hydra* bacterial symbiont, illustrated how symbionts undergo reductive specialization that reinforces dependency on host niches, shaping long-term stability of host–microbe interactions [18]. Three poster prizes were generously sponsored by Biomarker Technologies (BMKGENE). The first prize winner Yehor Tertyshnyk (University of Vienna, Austria) reported on the emergence of “entangled” genomic configurations in *Hydra vulgaris*. By studying the 3D genome with both sequencing (Micro-C) and imaging (DNA FISH) approaches, Yehor could show that TE-driven genome expansion presumably enhances ancestral regulation through functionally constrained chromosomal compartments. Second prize winner Clara Deleau (Sorbonne University, France) presented novel considerations on the evolution of striated muscles. The life cycle of their emerging model organism *Pelagia noctiluca* has fascinatingly lost the polyp stage, prompting the development of striated muscle tissue within a week of fertilization. Clara further found expression of certain genes that are homologs of known myogenic transcription factors in bilaterians, raising the question of whether striated muscle evolved twice independently, as previously thought, or may be homologous, as is the case with smooth muscle. The third prize winner Matthias Achrainer (University of Innsbruck, Austria) reported on a *Hydra* bioadhesive that lacks the large multidomain proteins common in other species. Rather, Matthias identified chitin and an uncharacterized secreted protein with an arabinose-binding domain as the core components of *Hydra* glue, distinct from other known adhesives.

## Conclusions and outlook

**Fig. 1:**
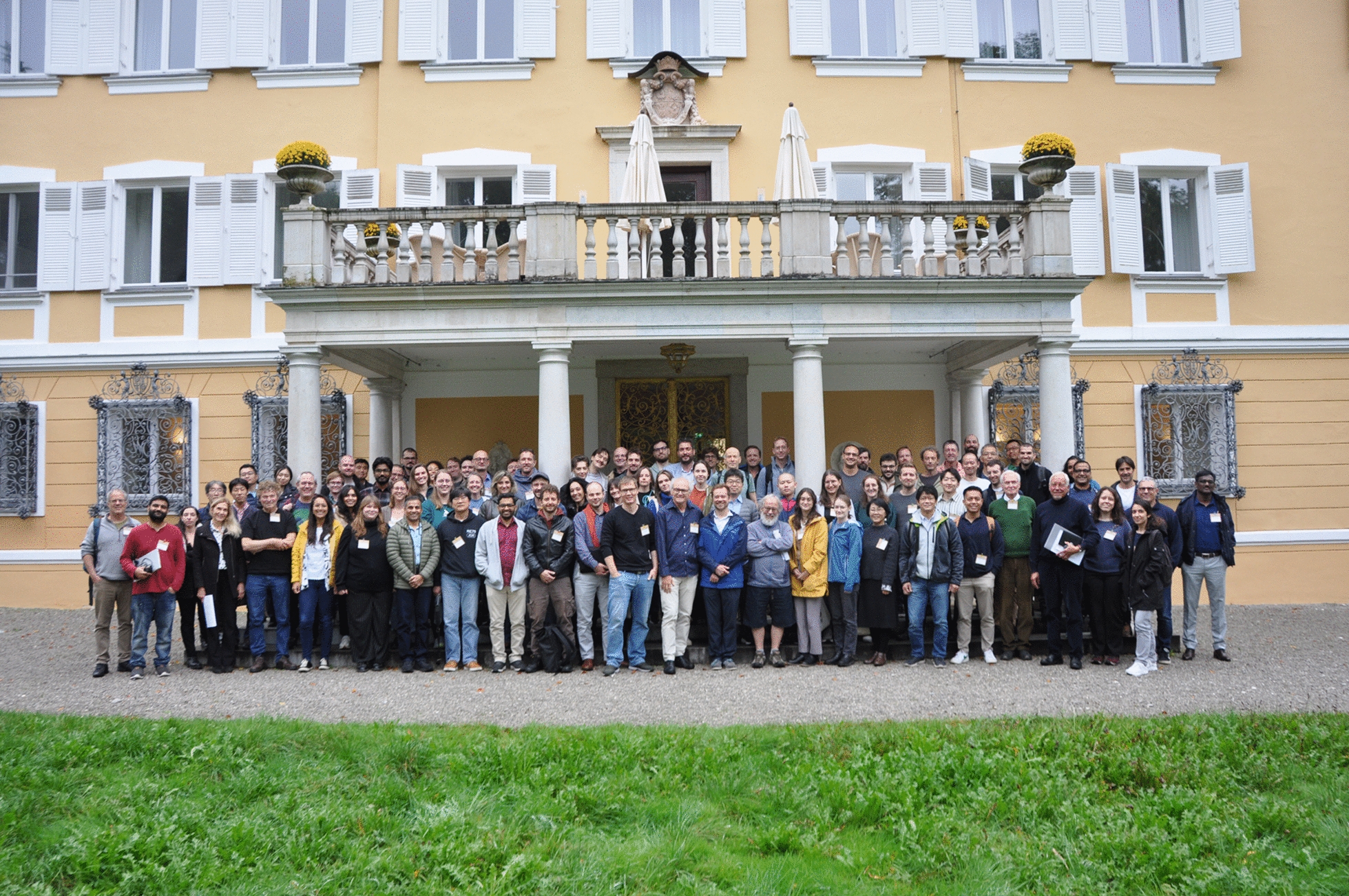
Group photo of all participants of the 2025 Tutzing International Workshop “Animal resilience and organismal response to environmental change: insights from basal metazoans” at the Evangelische Akademie, representing the diverse international community of researchers contributing to this year’s meeting

The 2025 Tutzing Workshop demonstrated the vibrancy of research on basal metazoans and their capacity to address fundamental evolutionary questions and contribute to the understanding of complex systems. Participants left with shared excitement and new plans for collaboration (Fig. 1). The meeting was universally regarded as a success, thanks in no small part to the unique setting of Tutzing, which facilitated both focused discussion and informal exchange. Several emerging themes were identified, such as the central role of transposable elements–once considered “junk”–as engines of evolutionary innovation in basal metazoans, the ‘unexpected’ complexity in “simple” systems, i.e., nervous and immune systems, in *Hydra* and *Nematostella*, and the importance of associated microbiomes as agents and mediators of host biology and resilience. Above all, the importance of open access, resource sharing, and working together by means of richly annotated publicly accessible datasets, adherence to common standards, and open communication and support to accelerate scientific insight was acknowledged. We are looking forward to an equally vibrant and inspiring meeting in 2027.

## Data Availability

Not applicable.
